# Draft Genome Sequence of Bacillus licheniformis Heshi-B2, Isolated from Fermented Rice Bran in a Japanese Fermented Seafood Dish

**DOI:** 10.1128/genomeA.00118-18

**Published:** 2018-03-08

**Authors:** Yu Kanesaki, Eri Kubota, Rumi Ohtake, Yukari Higashi, Junko Nagaoka, Tomonori Suzuki, Sayuri Akuzawa

**Affiliations:** a NODAI Genome Research Center, Tokyo University of Agriculture, Tokyo, Japan; b Research Institute of Green Science and Technology, Shizuoka University, Shizuoka, Japan; c Department of Nutritional Science and Food Safety, Tokyo University of Agriculture, Tokyo, Japan; d Department of Molecular Microbiology, Tokyo University of Agriculture, Tokyo, Japan

## Abstract

Bacillus licheniformis Heshi-B2 was isolated from fermented rice bran in Heshiko, a food produced by aging salted mackerel with fresh rice bran. Here, we report the draft genome sequence of B. licheniformis Heshi-B2, originating from a Heshiko sample from Fukui Prefecture, Japan.

## GENOME ANNOUNCEMENT

Fermented foods are produced using various functions of microorganisms. Heshiko, a traditional fermented seafood, is prepared with fresh rice bran and fish. Interestingly, the rice bran decomposes to an edible paste form after a fermentation process involving marine bacteria. Many marine bacterial species affecting microflora during Heshiko fermentation have been identified (1). In addition, a relationship between amino acid composition and the taste of fermented rice bran and the antithrombotic effects of Heshiko rice bran has been reported (2, 3). We isolated some bacteria from fermented rice bran in Heshiko, and analysis revealed that their draft genomes are from *Oceanobacillus picturae* Heshi-B3 (4) and *Paenibacillus amylolyticus* Heshi-A3 (5). Here, we report the draft genome sequence of a newly isolated bacterium from rice bran in Heshiko that belongs to the genus Bacillus. According its genetic similarity to other sequences, we named this strain B. licheniformis Heshi-B2.

Bacterial cells were cultured, and the cells were collected and treated with lysis buffer according to our previous methodology (4, 5). The bacterial genomic DNA was prepared using a DNeasy blood and tissue kit (Qiagen, Valencia, CA, USA). Five micrograms of genomic DNA was dissolved in Tris-EDTA buffer and prepared by sonication with a Covaris S-220 instrument (Covaris, Woburn, MA, USA) with an average size of 500 bases. The process of constructing the DNA library and sequencing the genome with the MiSeq platform (Illumina KK, Tokyo, Japan) followed those from our previous studies (4, 5). Sequenced reads were screened for quality (Phred score greater than 30) and were trimmed by 15 bases from the 5′ end and 2 bases from the 3′ end. The trimmed reads were assembled *de novo* using CLC Genomics Workbench version 9.5 (Qiagen, Valencia, CA, USA). The 76 assembled contigs (>1 kb) had a total length of 4,088,978 bp, an *N*_50_ value of 123,759 bp, and a GC content of 46.20%. Annotation of the contigs was performed using the DFAST pipeline (6). As a result, 4 rRNAs, 57 tRNAs, and 4,142 coding sequences were identified. So far, about 70 genome sequences of B. licheniformis have been deciphered. Our data will contribute to a better understanding of the genetic variation that exists among the halotolerant strains of B. licheniformis.

### Accession number(s).

The sequences and annotations of the 76 contigs of B. licheniformis Heshi-B2 have been deposited in DDBJ/ENA/GenBank under the accession number BEXU00000000. These are the original versions of the annotations.
